# Cervical Mucus Properties Stratify Risk for Preterm Birth

**DOI:** 10.1371/journal.pone.0069528

**Published:** 2013-08-01

**Authors:** Agatha S. Critchfield, Grace Yao, Aditya Jaishankar, Ronn S. Friedlander, Oliver Lieleg, Patrick S. Doyle, Gareth McKinley, Michael House, Katharina Ribbeck

**Affiliations:** 1 Department of Obstetrics and Gynecology, Division of Maternal Fetal Medicine, Tufts Medical Center, Boston, Massachusetts, United States of America; 2 Department of Biological Engineering, Massachusetts Institute of Technology, Cambridge, Massachusetts, United States of America; 3 MIT-Harvard Division of Health Sciences and Technology, Cambridge, Massachusetts, United States of America; 4 Department of Mechanical Engineering, Massachusetts Institute of Technology, Cambridge, Massachusetts, United States of America; 5 Department of Chemical Engineering, Massachusetts Institute of Technology, Cambridge, Massachusetts, United States of America; John Hunter Hospital, Australia

## Abstract

**Background:**

Ascending infection from the colonized vagina to the normally sterile intrauterine cavity is a well-documented cause of preterm birth. The primary physical barrier to microbial ascension is the cervical canal, which is filled with a dense and protective mucus plug. Despite its central role in separating the vaginal from the intrauterine tract, the barrier properties of cervical mucus have not been studied in preterm birth.

**Methods and Findings:**

To study the protective function of the cervical mucus in preterm birth we performed a pilot case-control study to measure the viscoelasticity and permeability properties of mucus obtained from pregnant women at high-risk and low-risk for preterm birth. Using extensional and shear rheology we found that cervical mucus from women at high-risk for preterm birth was more extensible and forms significantly weaker gels compared to cervical mucus from women at low-risk of preterm birth. Moreover, permeability measurements using fluorescent microbeads show that high-risk mucus was more permeable compared with low-risk mucus.

**Conclusions:**

Our findings suggest that critical biophysical barrier properties of cervical mucus in women at high-risk for preterm birth are compromised compared to women with healthy pregnancy. We hypothesize that impaired barrier properties of cervical mucus could contribute to increased rates of intrauterine infection seen in women with preterm birth. We furthermore suggest that a robust association of spinnbarkeit and preterm birth could be an effectively exploited biomarker for preterm birth prediction.

## Introduction

Preterm birth, or birth prior to 37 weeks of gestation, affects over 12% of pregnancies in the United States [1] and leads to $26 billion in annual healthcare costs [2]. Preterm birth is the leading cause of newborn mortality–more than half of all infant deaths in the United States occur to infants born prior to 32 weeks gestation [3]. Preterm infants that survive the newborn period are at increased risk for long-term health complications including neurodevelopmental disability, decreased growth, chronic lung disease and cardiovascular morbidity [4]–[7].

The pathophysiology most commonly linked to preterm birth is bacterial invasion from the colonized vagina to the sterile uterine cavity [8]–[10]. Common vaginal organisms, including genital ureaplasmas and mycoplasmas as well as gram positive and gram negative bacteria, are commonly found in amniotic fluid cultures of preterm birth patients [11]. In addition to infection, preterm birth is also associated with a robust inflammatory response in the amniotic fluid [12] and cervical mucus [13]. Taken together, these studies suggest that intrauterine infection occurs because the barrier to ascending infection is impaired. However, the barrier mechanisms preventing ascending intrauterine infection are poorly understood.

**Figure 1:**
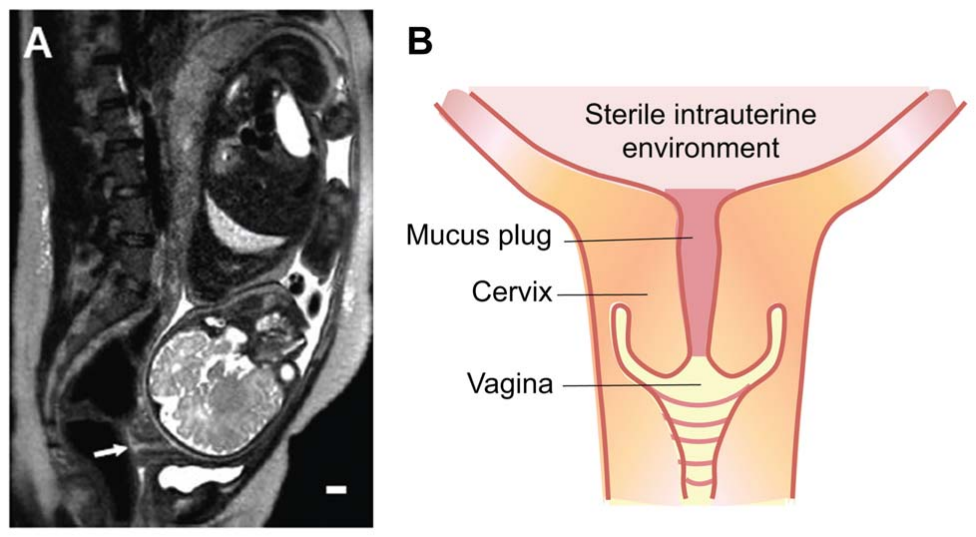
(A) MRI image of patient at 34 weeks gestation. Arrow points to cervical mucus plug. Scale bar: 1 cm. **(B) Diagram of the cervical mucus plug in situ.**

The main path of infection for vaginal microbes is the cervical canal, the main physical barrier that separates the vaginal tract from the sterile intrauterine cavity (Figure 1). A short cervix, as measured by transvaginal ultrasound, is a strong predictor of subsequent preterm birth [14], [15] and is typically followed by treatment strategies such as progesterone supplementation or cerclage placement [16]–[19]. The cervical canal is filled with mucus, a ubiquitous hydrogel that lines all wet surfaces in the body including the respiratory and gastrointestinal tracts. Its main gel-forming constituents are the mucin glycoproteins, which entangle to form a viscoelastic hydrogel. The mucus barrier functions to both shield the underlying epithelia from infectious agents and other environmental particles/pathogens while allowing selective passage to nutrients, ions, gases, proteins and sperm. Cervical mucus is secreted from goblet cells within crypts lining the cervical canal [20], [21] and, during pregnancy, condenses to form a large compact structure commonly known as the “cervical mucus plug” that is shed prior to delivery [22], [23]. The cervical mucus plug in normal pregnancy has properties of both innate and adaptive immunity [24]–[26], and is, therefore, thought to play a vital protective role during pregnancy [23]. However, a detailed understanding of the barrier properties of cervical mucus is not understood.

Given the role of cervical mucus as a selectively permeable barrier, its location between the colonized vagina and the sterile uterine cavity, and the strong association between intrauterine infection and preterm birth, we sought to examine changes in the barrier properties of cervical mucus in women at high and low-risk for preterm birth. We hypothesize that in high-risk pregnancies, cervical mucus has altered biophysical and biochemical properties, allowing for increased ascension, proliferation, or virulence of bacteria, which can ultimately trigger preterm delivery. To test this hypothesis we used two different rheological techniques to evaluate the extensional and viscoelastic properties of the cervical mucus samples. In addition, mucus permeability was directly visualized by the passage of fluorescent microbeads through mucus samples.

## Materials and Methods

### Patient Selection

Internal Review Board (Tufts Medical Center) approval was obtained prior to the start of this pilot case-control study (IRB#9355). Written informed consent was obtained from all participants prior to enrollment. Patients at high-risk for preterm delivery were defined as: 18–50 years old with singleton pregnancies between the gestational ages of 20 weeks 0 days and 34 weeks and 0 days gestation who were admitted to a tertiary care facility with suspected preterm labor. For the purposes of this study, preterm labor was defined as documented cervical change (dilation and/or effacement) in the setting of regular uterine contractions and 2 cm or greater cervical dilation. Patients at high-risk for preterm birth were approached to participate after uterine contractions abated and no cervical exam was performed for 48 hours. Hence, no patient was in active labor and the high-risk patients in the study are more properly described as being in ‘arrested preterm labor.’ Exclusion criteria included significant maternal medical conditions predisposing the patient to preterm labor (i.e. collagen disorder, systemic infection), a maternal history of smoking or drug abuse at any time, a history of abnormal pap smear or cervical procedure at any time, amniocentesis or chorionic villus sampling within four weeks of enrollment, placenta previa during the incident pregnancy, rupture of amniotic membranes or active vaginal bleeding at the time of sample collection, a documented vaginal infection (yeast, chlamydia, trichomoniasis, gonorrhea, bacterial vaginosis) or urinary tract infection within 6 months, intra-abdominal surgery during the incident pregnancy and a pelvic exam, intercourse or antibiotic use within 48 hours of sample collection.

High-risk patients were compared to gestational age-matched control subjects (1:1) who were not experiencing preterm labor. Low-risk control subjects were recruited from the antepartum service or the outpatient clinic. Recruitment occurred between February 1^st^ 2011 and September 1^st^ 2012. Guidelines for the reporting of observational studies in epidemiology (STROBE) were followed [27].

### Cervical Mucus Sample Collection

Cervical mucus samples were obtained by sterile speculum exam using a Cooper Surgical Endocervical Curette with a 12cc Vacu-Lok Syringe placed at the external cervical os. Cervical mucus samples were used immediately or snap frozen in liquid nitrogen and stored at −80°Celsius. Patient samples were de-identified and data was stored in a password protected database. Due to the difficulty of extracting cervical mucus, we could obtain only relatively small sample volumes (approximately 200 µL). This volume was not sufficient to conduct each of the presented assays. Hence, the number of mucus samples that were used per assay was lower than the number of enrolled participants.

### Extensibility Measurement and Shear Rheometry

To elucidate differences in extensional rheology, a Capillary Breakup Extensional Rheometer (CaBER) [28] was used. The CaBER draws a material apart into a filament at a fixed rate for rheological observation and the determination of spinnbarkeit (capacity to form filaments) [29]. 200±100 μL of cervical mucus sample was placed between two circular metal plates that were 6 mm in diameter, with an initial gap of 2 mm. The plates were then separated to a distance of 20 mm (maximum separation distance attainable with our CaBER device) at a constant rate of 3.6 mm/s and the separation distance at which the sample broke (“break point”) was recorded. For statistical calculations, a “break point” of 20 mm was used for those samples which remained intact.

Shear rheometry was performed with a TA instruments ARG2 controlled stress rheometer. Approximately 75 µL of cervical mucus was placed in a 1.5 mm gap between an 8 mm diameter steel plate, and a Peltier plate whose temperature was controlled at 25°C. During the test, we apply a sinusoidally varying strain to the mucus sample, and measure the resulting stress response. The storage modulus G′ (storage modulus, quantifying the elastic, solid-like, recoverable property of a substance) and the loss modulus G′′ (loss modulus, quantifying the viscous, liquid-like, non-recoverable property) are determined from this stress response. A perfectly viscous material would have G′ = 0, while a perfectly elastic material would have G′′ = 0. The upper plate was oscillated at a strain amplitude of 1%. It was ensured that this strain amplitude was in the linear viscoelastic regime for both mucus samples.

### Measurement of Mucus Permeability

To study permeability of the native cervical mucus, a bead translocation assay was performed with streptavidin-activated glass slides as reported [30]. Prior to the experiment the slides were pre-incubated for 30 minutes in 0.5% BSA to eliminate non-specific binding, and encased in an Arrayit 24-well multiplex microarray cassette. 25 μL of mucus sample, or 20 mM HEPES buffer without mucin, were spread in triplicate in individual wells. 5 uL biotinylated Fluospheres (0.2 μm, Invitrogen) at a concentration of 5×10^6^ particles/mL was added on top the mucus or buffer and allowed to diffuse for 2 hours at room temperature. The glass slides were washed of the mucus three times in washing buffer, with 0.1% Triton-X detergent added to the first washing step. Next, three images per well were acquired with a fluorescence microscope at 10× to quantify the number of streptavidin-bound biotin beads that had passed through the mucus to the underlying surface. The mean of the nine images per sample was taken to represent the number of beads that passed through each sample. The investigator responsible for quantifying the number of beads passing through each sample was blinded to the cervical mucus sample study group.

### Scanning Electron Microscopy (SEM)

Mucus samples were collected and directly placed in mucin wash buffer (0.02% saponin and 1.0% ruthenium red in cacodylate buffer 0.1 M at pH 7.4). These samples were stored at 4°C until further processing (1–2 days). Mucus samples were placed in fixation buffer (mucin wash buffer plus 3.0% glutaraldehyde) and fixed overnight at room temperature. This was followed by four washes in mucin wash buffer (25 min. each), and then secondary fixation in mucin wash buffer plus 1.0% OsO_4_ for 2 h. Samples were then treated with 1.0% thiocarbohydrazide in water for 20 min., then 1.0% OsO­_4_ for one hour. These two steps were repeated, and the samples were then washed four times with deionized water for ten min. each. After ethanol series dehydration (25%, 50%, 75%, 95%, 100%, 100%, 100% ethanol for 20 min. each), samples were critical point-dried and mounted on aluminum stubs using silver colloid paint. Samples were imaged on a Zeiss Supra 55VP FE-SEM using a secondary electron Everhart-Thornley detector.

### Statistical Analysis

Two tailed paired and unpaired Student's T-tests and Chi-square test were performed where appropriate to determine differences between patient groups. P-values <0.05 were considered significant.

## Results

### Participants

A total of forty four patients were enrolled. Two samples were subsequently discarded for exclusion criteria violation noted after collection. Samples from the initial six patients were used for optimization of the permeability assay. Subsequently, a total of 36 patients (18 high risk, 18 low risk gestational age matched controls) were available for analysis (Table 1). Due to the relatively small amount of cervical mucus available from each participant (approximately 200 µL), individual samples were not available for use in all assays. Among high-risk patients, cervical dilation was significantly increased compared with women at low-risk for preterm delivery (2.8±0.8 cm vs. 0.00±0.0 cm, p<.01). The demographics of the high-risk and low-risk patients were similar, but we observed more African-American patients in the low-risk cohort. Less cervical mucus was collected from low-risk patients, likely due to the limited access of the closed cervix and the thickened consistency of the mucus. In evaluation of the clinical outcome, the high-risk patients delivered earlier compared to low-risk controls (34.4±4.3 weeks versus 37.1±2.1 weeks, p<.05). While there was a significant difference in delivery gestational age between the two groups, we noted that some low-risk patients also delivered at or before 37 weeks of gestation. The reason for this is that several of the ‘low-risk’ patients who were recruited from the inpatient antepartum service had risk factors for indicated preterm delivery (i.e. preeclampsia). Hence, the frequency of indicated preterm birth was increased in the ‘low-risk’ cohort, which resulted in a lower than expected mean delivery gestational age for this cohort (37 weeks).

**Table 1:** Patient Characteristics.

Characteristic	High Risk (n = 18)	Low Risk (n = 18)	P–value
Age (Years)	27.4 (+/−6.6)	29.6 (+/−5.8)	0.36
Gravidity	2.7 (+/−1.6)	3.0 (+/−1.9)	0.71
Parity	1.0 (+/−1.1)	0.7 (+/−0.8)	0.50
Race (%)			<0.001
White	22	50	
Black	0	11	
Hispanic	50	28	
Other	28	11	
Gestational Age (wks)	30.8 (+/−3.4)	30.4 (+/−3.3)	0.74
Dilation (cm)	2.8 (+/−0.8)	0.00 (+/−0.0)	<0.001
Prior PTB (%)	22	16	0.64
Positive GBS carrier (%)	17	17	1.0
Mucus collected (µl)	264.0 (+/−117.0)	192.0 (+/−61.5)	0.018
Gestational Age at Delivery (wks)	34.4 (+/−4.3)	37.1 (+/−2.8)	0.03

Expressed as Mean (+/− SD) or percentage; GBS: Group B Streptococcus.

### Extensional rheometry reveals a high spinnbarkeit for cervical mucus in patients at high-risk of preterm birth

**Figure 2:**
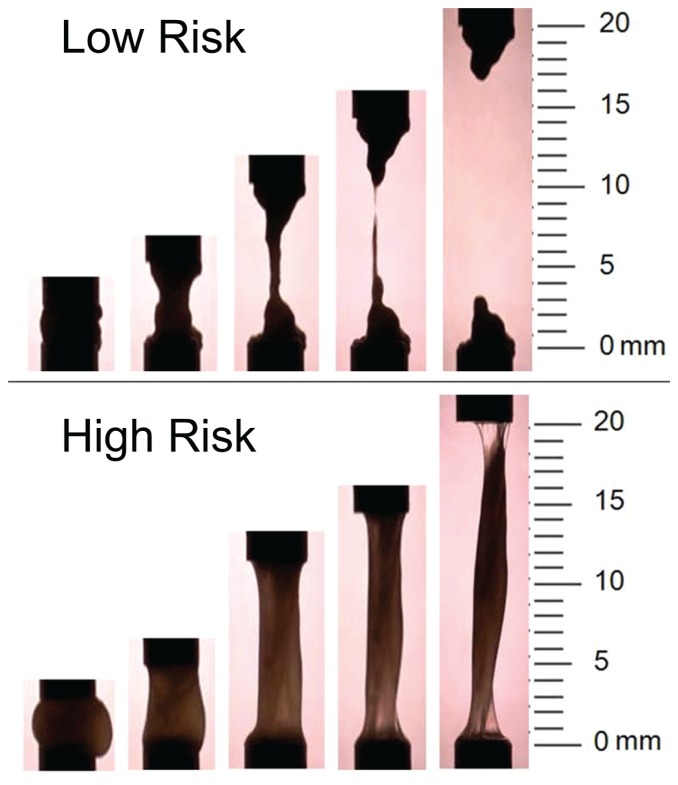
Example time series of spinnbarkeit test at 2, 5, 10, 15, and 20 mm in low-risk and high-risk cervical mucus samples. Almost all high-risk samples could be stretched to at least 20 mm without breaking (exhibiting spinnbarkeit). In contrast, mucus from low-risk patients had an average break length of 13.8±2.4 mm.

During collection of the cervical mucus we observed a marked visual difference between mucus from women at low-risk and women at high-risk for preterm birth: control mucus from low-risk women was homogeneously opaque and paste-like, while mucus from high-risk women was partially translucent, with a texture resembling raw egg white. We postulated that there was a disparity in elasticity, which was investigated using a Capillary Breakup Extensional Rheometer (CaBER). [29]. Cervical mucus from four high-risk and four gestational age matched low-risk controls were used. This experiment revealed a significant difference in cervical mucus break point between high-risk and low-risk samples (mean (SD), 19.5 mm (±1.0 mm) vs. 13.8 mm (±2.4 mm); p<0.01). 3 out of 4 high-risk samples remained intact at 20 mm after plate separation and clearly displayed spinnbarkeit, a phenomenon which is reminiscent of cervical mucus during ovulation and should be absent in pregnancy [23]. In contrast, none of the low-risk samples remained intact (Figure 2).

### Shear rheometry reveals a higher elasticity of high-risk mucus than low risk mucus

**Figure 3:**
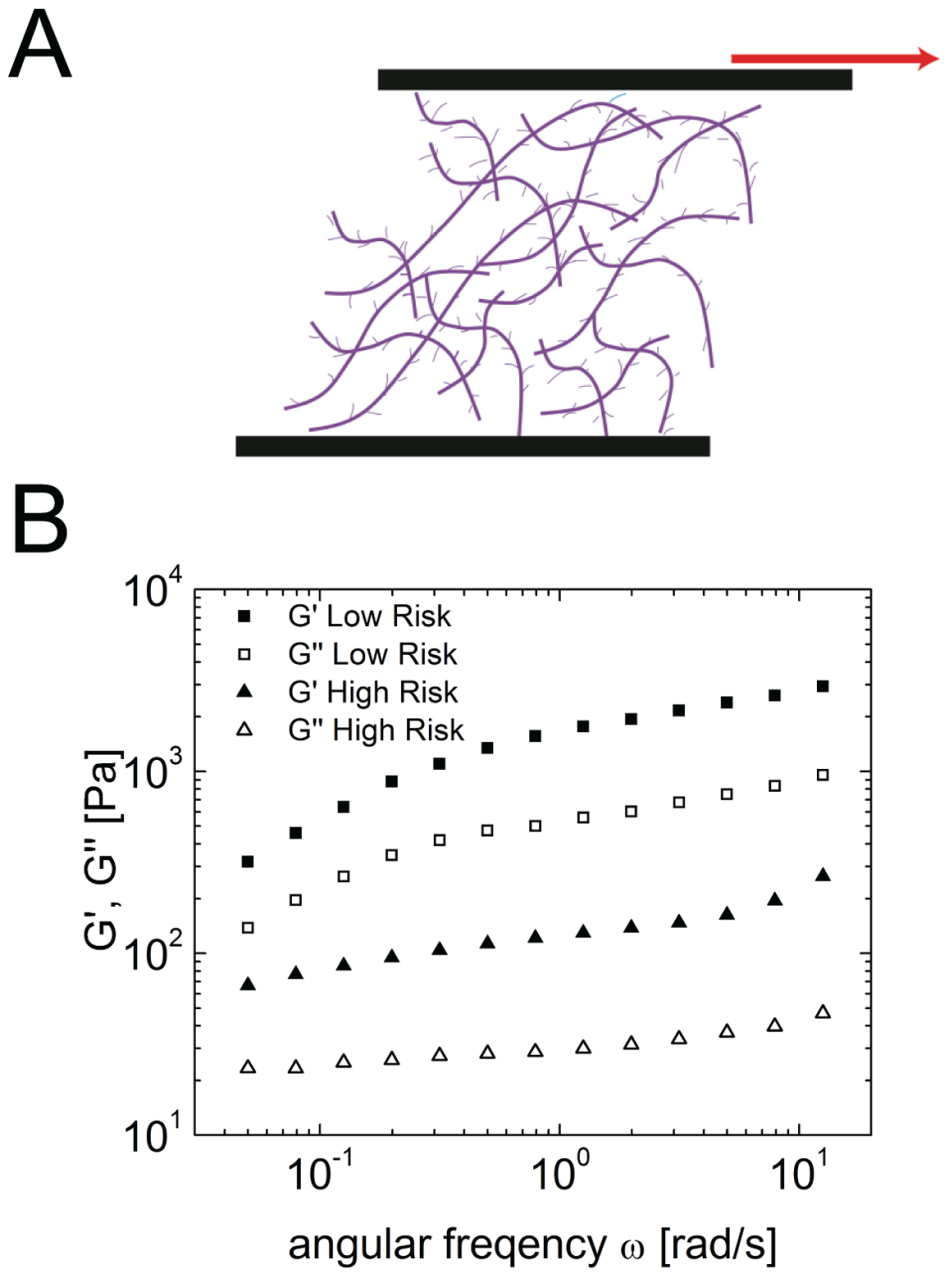
(A) Diagram of shear rheology. Rotational shear force applied to cervical mucus sample. **(B) Example linear viscoelastic spectra of high risk and low risk cervical mucus samples.** Storage modulus G′ and loss modulus G” of low risk mucus is an order of magnitude greater than that of high-risk mucus, indicating that high-risk mucus is more weakly cross-linked than low-risk mucus.

Since rheological properties are key determinants of hydrogel barrier functions [31], we performed a more detailed rheological characterization of cervical mucus from high-risk and low-risk patients. Specifically, we investigated the viscoelasticity of the mucus samples of three high-risk and three gestational age matched controls using a rotational shear rheometer, measuring G′ (storage modulus) and G′′ (loss modulus). Across all pairs, both high-risk and low-risk mucus had a higher storage modulus than loss modulus, indicating that all cervical mucus samples are more solid-like than liquid-like. In addition, both the storage and loss moduli of cervical mucus samples from patients at high-risk of preterm delivery were found to be an order of magnitude lower than that of cervical mucus samples from gestational age matched low-risk controls (Figure 3). This result suggests that the gel-forming mucins (and potentially other molecules) within high-risk mucus may be less effectively cross-linked, thereby generating a weaker gel, possibly with larger pores.

### Scanning Electron Microscopy reveals heterogeneity within mucus samples

**Figure 4:**
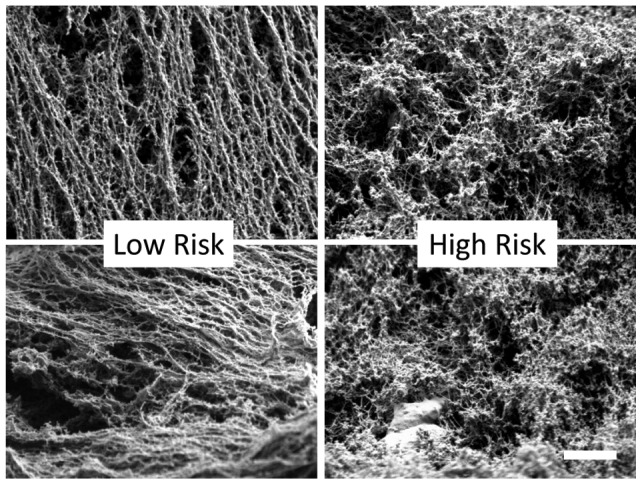
(A) Diagram of bead permeability assay. (B) Bead permeability assay results. High risk cervical mucus samples displayed significantly more permeability to the biotin labeled polystyrene beads compared to low risk controls (5.6 beads/field (+/−2.6) vs. 2.2 beads/field (+/−1.2), p = 0.006).

In an attempt to directly visualize the mucin cross-linking, Scanning Electron Microscopy (SEM) was performed on two high-risk and two low-risk gestational age matched controls (n = 4). The sample preparation resulted in dehydrated, brittle samples, which were fractured prior to imaging. We noted a high degree of heterogeneity within each sample, but we imaged regions of filamentous networks in matched locations (subsurface regions along fractures) for comparison between samples. In these matched regions, we noted that low-risk samples tended to have thicker filaments (we interpret these to be collapsed bundles of filaments), whereas high-risk samples had thinner, but less collapsed filaments. The increase in filament collapse among low-risk samples is indicative of an increased retraction force, which is consistent with a high degree of crosslinking. A thickening of cervical mucus during pregnancy, which may be related to the increased degree of crosslinking detected here, has been observed previously [32]. Nonetheless, the small sample size and the heterogeneity of the samples left us unable to draw distinct conclusions regarding visualized differences among samples (Figure 4).

### The permeability of cervical mucus from women at high-risk pregnancy appears increased

**Figure 5:**
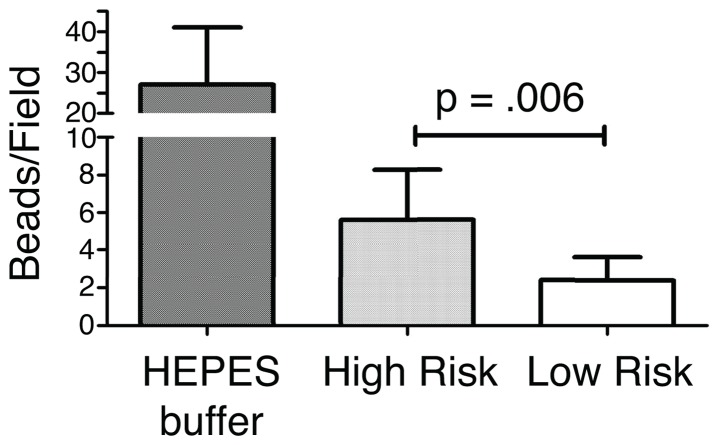
Example scanning electron microscopy images. Cervical mucus samples from low-risk and high-risk patients were fixed and dehydrated for examination by electron microscopy. Scale bar: 200 nm.

To measure the permeability of healthy and preterm pregnancy mucus we performed a translocation assay in 24-well multiplex microarray cassettes containing streptavidin-coated glass slides. Each well was filled with mucus followed by biotinylated fluorescent polystyrene microspheres. After two hours of incubation, the number of streptavidin-bound biotin beads that had passed through the mucus to the underlying surface was quantified. Taking the average of nine gestational age matched pairs of high-risk and low-risk samples (n = 18), we found that those samples collected from patients at high-risk of preterm delivery showed more permeability to the biotin labeled polystyrene beads (5.6 beads/field (+/−2.6) vs. 2.2 beads/field (+/−1.2), p = 0.006) (Figure 5). The mean ratio in paired samples (high-risk/low-risk) was 2.7 (+/−1.4; p = 0.006, 95%CI 1.2–5.2). These data suggest suggests that high-risk pregnancy mucus is more easily penetrated by particles than mucus in normal pregnancy conditions.

## Discussion and Conclusions

Here we show that cervical mucus from women at high risk for preterm birth is more translucent, extensible, and permeable compared to cervical mucus from patients at low risk of preterm birth. While optical properties and spinnbarkeit are more easily discernible, permeability is clinically significant, as it can allow for an increased number of foreign particles such as viruses or bacteria to harmfully traverse the barrier of the cervical mucus plug. We hypothesize that in high-risk pregnancies, cervical mucus fails to develop into the thickened and impermeable "pregnancy state", allowing for increased ascension of bacteria, which is a known cause of preterm delivery [33]. A molecular dissection of high-risk mucus is now needed, which will likely provide insight into the causes of altered cervical mucus properties and direct the design of intervention strategies.

The results of this study showed that altered mucus biophysical properties are associated with an increased risk of preterm birth. However, due to the specific design of this case-control study it is not possible to determine if altered cervical mucus is a primary cause for the cascade of events leading to preterm birth, or whether it is the consequence of different pathological processes. An important task for future studies is to distinguish between these two possibilities. Further limiting our study is the relatively small numbers of patients. Nevertheless, we regard this pilot study as an important first step to a more comprehensive understanding of the cervical mucus properties in relation to preterm birth.

**Figure 6:**
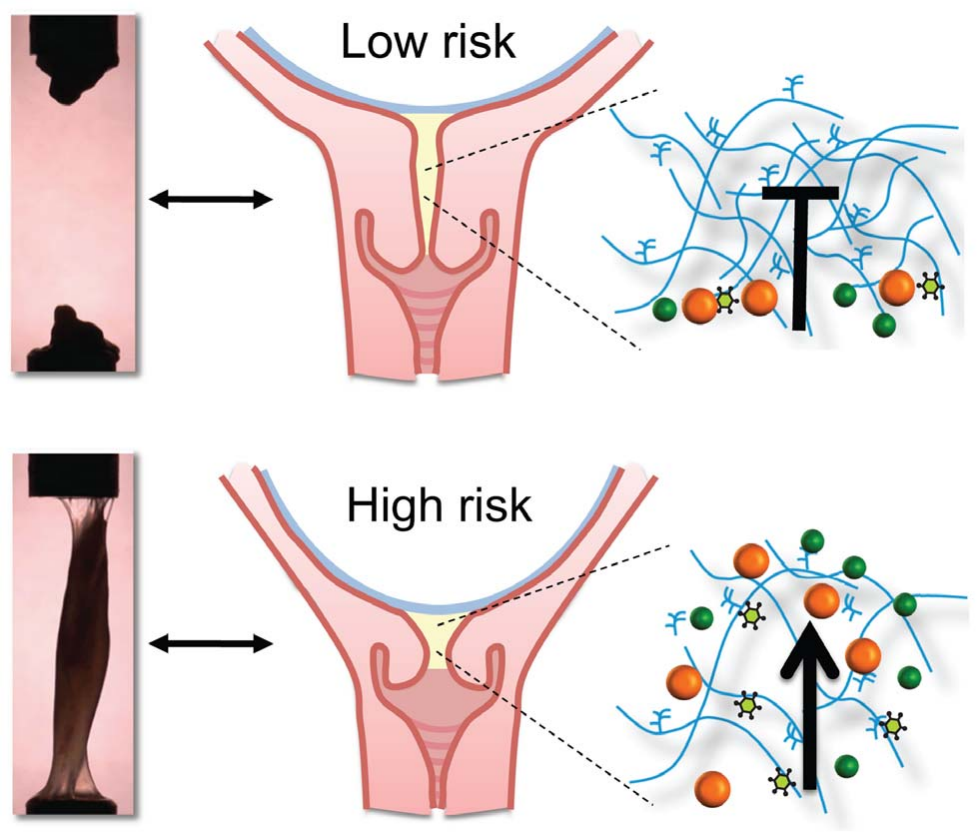
Summary. In women at high risk of preterm birth (with a short and dilated cervix), we find that the cervical mucus does display spinnbarkeit, is more weakly cross-linked and is a less effective barrier.

One primary function of cervical mucus is to prevent microbial ascension into the uterine cavity (Figure 6) [23], but its role could be more far reaching than this. Work by Mysorekar showed that the basal plate of the placenta is not always sterile, but instead colonized by intracellular bacteria in 27% of uncomplicated term vaginal deliveries, increasing to 55% in spontaneous preterm deliveries that occur prior to 28 weeks [34]. Further complicating the local environment is the presence of microbial biofilms, which can be present at the internal cervical os in women delivering preterm [35]. Hence, infection mediated preterm birth might also be caused by unchecked proliferation or virulence of preexisting microbes, processes which may be the result of altered or dysfunctional cervical mucus properties. These previous observations, together with the present study, urge better understanding of the mechanisms that lead to microbial passage and proliferation in the cervical mucus environment.

Prior work on cervical mucus from pregnancy has focused on the antimicrobial [24]–[26] and proinflammatory [36], [37] mediators in women at high-risk vs. low-risk for preterm birth. However, the barrier function of the mucus hydrogel depends not only on molecular mediators but also on the physical and mucoadhesive properties of the mucin fibers [31]. Studies of non-pregnant cervical mucus suggest that the pore size could be altered by controlling hydrophobic interactions between the mucin fibers [38]. Movement of viral particles in cervical mucus was governed not by steric obstruction but by the mucoadhesive properties of the fibers [39]. In addition, particle translocation through a mucus layer is known to be influenced by other factors such as pH and ionic concentration [31], [40]. The data from this study support the concept that changes in the physiochemical properties of the mucus fibers have a direct effect on mucus permeability. A comprehensive investigation of cervical mucus barrier properties is needed to fully elucidate mucus barrier function during pregnancy.

The rheological characteristics of mucus from high-risk patients were qualitatively similar to the characteristics of ovulatory cervical mucus, which has been documented to be thin, translucent, less acidic, and exhibit spinnbarkeit [25], [32]. Due to an increase in estrogen, ovulatory cervical mucus water content increases from 96% to over 97.5%, with a strong correlation noted between hydration, viscosity, and sperm penetrability [36], [37]. Shortly after conception, the cervical mucus meshwork tightens, forming the dense cervical mucus plug [32]. Under the influence of progesterone, cervical mucus becomes scant, thick, acidic, drier, and more viscous [21], [22], [39], [41]. It also becomes more opaque and spinnbarkeit is absent [38]. Given the similarities in the rheological characteristics between ovulatory mucus and mucus from high-risk patients, it is not surprising that mucus from high-risk patients was more permeable compared with mucus from low-risk patients.

A robust association of spinnbarkeit and preterm birth could be an effectively exploited biomarker for preterm birth prediction. In clinical medicine, there is intense interest in finding biomarkers to predict preterm birth. Currently, measurement of fetal fibronectin and cervical length screening are used to predict preterm birth [15], [41]. However, many preterm births occur in women without risk factors, which has prompted a largely disappointing search for other biomarkers to predict preterm birth [42]. Just as midcycle spinnbarkeit is used to aid family planning, it is conceivable that spinnbarkeit could be incorporated into a program of preterm birth risk assessment. Improved preterm birth prediction will aid targeting of therapeutic interventions for high-risk women and avoid therapy for low-risk women.

The cervical mucus is an attractive therapeutic target for prevention of preterm birth. Using clinical and sonographic variables, it is possible to identify a cohort of patients who are candidates for therapy [43]. Indeed, vaginal application of progesterone is currently used to prevent preterm birth in the setting of a short cervix [17], [44]. In non-pregnant women, cervical mucus viscosity and elasticity have been correlated with improved host defense against upper genital tract infections [43], [44]. In pregnant women, the development of the cervical mucus plug correlates with mesh tightening as observed on SEM, which likely aids host defense [25], [32]. Future development of an ‘engineered’ cervical mucus could include localized gene delivery [45], sustained release of antibiotics [46], optimized mesh spacing [47] and surface qualities [48].

In summary, among women at high-risk for preterm birth, we found that cervical mucus was more permeable compared with low-risk women. This increased permeability correlated with changes in extensional rheology and viscoelastic properties. While it is not yet possible to determine if these changes are a cause or a consequence of preterm labor, these altered cervical mucus properties could relate to the increased incidence of microbial invasion of the uterine cavity seen in preterm birth. Future research will probe the mechanisms that lead to the alterations in cervical mucus properties. If changes in cervical mucus are indeed causal, future work could then move forward with a view towards designing novel intervention strategies aimed at the cervical mucus plug.
